# Targeting resources efficiently and justifiably by combining causal machine learning and theory

**DOI:** 10.3389/frai.2022.1015604

**Published:** 2022-12-07

**Authors:** Ozden Gur Ali

**Affiliations:** College of Administrative Sciences and Economics, Koç University, Istanbul, Turkey

**Keywords:** causal machine learning, interpretability, explainable AI, heterogeneous treatment effects, monotonicity constraints, public health, efficient resource allocation, GLM_QC

## Abstract

**Introduction:**

Efficient allocation of limited resources relies on accurate estimates of potential incremental benefits for each candidate. These heterogeneous treatment effects (HTE) can be estimated with properly specified theory-driven models and observational data that contain all confounders. Using causal machine learning to estimate HTE from big data offers higher benefits with limited resources by identifying additional heterogeneity dimensions and fitting arbitrary functional forms and interactions, but decisions based on black-box models are not justifiable.

**Methods:**

Our solution is designed to increase resource allocation efficiency, enhance the understanding of the treatment effects, and increase the acceptance of the resulting decisions with a rationale that is in line with existing theory. The case study identifies the right individuals to incentivize for increasing their physical activity to maximize the population's health benefits due to reduced diabetes and heart disease prevalence. We leverage large-scale data from multi-wave nationally representative health surveys and theory from the published global meta-analysis results. We train causal machine learning ensembles, extract the heterogeneity dimensions of the treatment effect, sign, and monotonicity of its moderators with explainable AI, and incorporate them into the theory-driven model with our generalized linear model with the qualitative constraint (GLM_QC) method.

**Results:**

The results show that the proposed methodology improves the expected health benefits for diabetes by 11% and for heart disease by 9% compared to the traditional approach of using the model specification from the literature and estimating the model with large-scale data. Qualitative constraints not only prevent counter-intuitive effects but also improve achieved benefits by regularizing the model.

## Introduction

Efficient resource allocation requires identifying those groups/individuals with high benefits (treatment effect) per unit cost (Haupt and Lessmann, 2022). For example, economic incentives in motivational tools built into health wearables constitute a treatment to improve healthy behaviors and health outcomes (Adjerid et al., 2022). Accurately estimating heterogeneous treatment effects allows focusing the limited resources on individuals whose health would benefit the most from these incentives and thus increases the targeting efficiency.

The gold standard for estimating causal effects is randomized controlled experiments. In their absence, causal effects can be estimated with observational data, providing that the model is correctly specified and includes all confounding covariates (Gelman and Hill, 2006). When used with large amounts of data that contain all confounders, new *causal machine learning* (*C-ML*) methods (e.g., causal forest, BART, X-Learner) learn the non-parametric model structure and estimate heterogeneous treatment effects accurately (Hill, 2011; Athey et al., 2019; Künzel et al., 2019; Gubela et al., 2020). On the other hand, their complexity makes these models uninterpretable.

In general, black-box models face lower acceptance from the decision-makers, who view them skeptically and compare them to their own understanding (Žliobaite et al., 2016; Coussement and Benoit, 2021). Decision-makers will want to see that the model is qualitatively in line with the current understanding and that the new findings are reasonable considering other external evidence. Transparency and fairness of decisions to determine which programs should receive investment are important in institutional settings, particularly in the public sector (Bilkey et al., 2019).

Further increasing the importance of interpretability, unlike the “traditional” machine learning prediction tasks for an observable quantity, such as whether the person has the disease or not, causal machine learning methods estimate the *unobservable* treatment effect, e.g., change in the disease probability due to increased physical activity, and therefore lack an objective accuracy measure. Causal inference with observational data necessarily depends on the external assumptions (Athey, 2017). Hence, whether the model “makes sense” is not only important for organization acceptance, but it also serves as a diagnostic about whether the model is overfitting or has confounding problems. Justifiability requires interpretable models that can be related to the existing understanding of the subject.

The key questions we seek to address in this paper are “how can we learn heterogeneous treatment effects (HTEs) accurately without losing interpretability and connection to existing theory?,” and “how can we facilitate a good tradeoff between targeting efficiency and interpretability?”.

Our contributions are as follows: first, we propose a methodology to incorporate causal machine learning findings into a theory-driven model specification for HTE estimation. To the best of our knowledge, this is the first use of Causal ML and Explainable Artificial Intelligence (XAI) to identify the moderators of the causal (treatment) variable and functional form of the effect. Second, we introduce the generalized linear model with qualitative constraint (GLM_QC) method to efficiently impose sign and monotonicity constraints on the main and two-way interaction effects of discretized variables. Third, we provide empirical evidence that enhancing a theory-driven model with additional heterogeneity dimensions and qualitative constraints based on Causal ML insights increases the targeting efficiency. Fourth, we show that imposing sign and monotonicity constraints leads to more accurate HTE estimates compared to allowing arbitrary non-linear effects and prevents counter-intuitive effects.

The case study deals with estimating the HTE of increasing physical activity on Diabetes and Ischemic Heart Disease (IHD) probability among the adult population in Turkey. The objective is to maximize the health benefits, by targeting the limited public health resources to encourage individuals to increase their physical activity. The observational data consist of five waves of the biannual individual level nationally representative health surveys conducted by the Turkish Statistical Institute (TUIK) with more than ninety thousand observations, providing self-reported socioeconomic, demographic, behavior, and disease information. The theory-driven initial model terms and the qualitative constraints on sign and monotonicity are taken from the literature (Ali et al., 2020).

The rest of the paper is organized as follows. The next section provides an overview of related work in treatment effect estimation, sign and monotonicity constraints in regression, causal machine learning methods, and XAI, with an emphasis on tools used in the study. The problem and the methodology section lays out the proposed methodology, including GLM_QC. The case section describes the case study, the data sources, and the theoretical specifications. Results section applies the methodology to the case and provides insights and performance comparison with the benchmarks. We conclude with managerial and methodological implications and limitations.

## Related work

### Treatment effect

The causal effect is the difference between a certain outcome and a “counterfactual” obtained under a different treatment, such as disease probability with the current and one higher level of physical activity, or customer churn probability with and without a retention offer.

While controlled randomized experiments are the gold standard to guard against confounding and eliminate selection and self-selection bias in the data, they are frequently infeasible or not cost-effective. The Neyman-Rubin potential outcome model (Rubin, 1974) allows for causal effect estimation in observational studies with two main assumptions: (a) the treatment of an individual will not affect the outcome of others and the treatment *w* is standard; (b) unconfoundedness, indicating that the treatment assignment is independent of the outcomes, given the covariates *X*, that is, *y*⊥*w*|*X*, typically extended with the overlap (or common support) assumption that all individuals have a non-zero probability of treatment. The unconfoundedness assumption ensures that selection bias is zero conditional on the confounding covariates (Gelman and Hill, 2006; Athey and Imbens, 2016). Ho et al. (2017) discusses the methods to infer treatment effects in case of a concern for endogeneity, e.g., due to the omission of confounding variables from the model, including instrumental variables, regression discontinuity, and differences in different approaches.

In this study, we focus on the methods that rely on the above two assumptions to estimate heterogeneous treatment effects.

*Regression* of *y* with *w* and all confounders can be used to estimate causal effects, assuming the model is correctly specified (including linearity and interactions) (Gelman and Hill, 2006). The treatment effect, τ(*X*), is defined as follows, where ω_0_ and ω_*s*_ are the control and treatment levels, respectively.


$$\begin{array}{l} \tau \left(X\right)=E\left[y|w=\omega_{s},X\right]-E\left[y|w=\omega_{0},X\right] \end{array}$$

Unlike the regression model, which is global, *matching* relies on the local estimates by comparing the outcome of treated individuals with those of similar ones who are untreated. While nearest neighbor matching is popular due to its simplicity (Stuart, 2010), an alternative is kernel smoothing, which averages the outcomes in the entire control group by weighting the subjects according to their closeness (Su et al., 2012). *Propensity score* methods use the conditional treatment probability of the individual, *p*(*w*|*X*), to estimate treatment effects. The scores can be estimated using any supervised machine learning technique such as logistic regression, support vector machines, neural networks, or random forests (Westreich et al., 2010). They can be used to weight observations in calculating average treatment effects (ATEs) to adjust for over-/under-representation among other uses.

While earlier research focused on ATE in a population, the availability of large-scale data and advanced statistical and machine learning methods enabled the detection and measurement of heterogeneous (or Individual) treatment effects (HTEs), denoted by τ_*i*_.


$$\begin{array}{l} \tau_{i}=E\left[y_{i}|\;{w_{i}=\omega }_{s},X_{i}\right]-\;E\left[y_{i}|{w_{i}=\omega }_{0},X_{i}\;\right]. \end{array}$$

Operation management and marketing applications such as uplift modeling, differential response modeling, incremental response, true-lift, or net modeling seek to identify the individuals or segments with high treatment impact for efficient use of resources with targeting (Ascarza, 2018). Uplift models for retention “recognize customer heterogeneity with respect to the incremental impact” of the treatment (Ascarza et al., 2018). Gubela et al. (2020) provides a review and evaluate supervised machine learning algorithms in the context of revenue uplift modeling for marketing campaigns.

### Heterogeneous treatment effect learning in healthcare

In healthcare applications, quantifying heterogeneous treatment effects allows for taking appropriate preventive measures and personalizing treatments. While it is possible to learn the causal effects and calculate counterfactual outcomes with sufficiently variant observational data, it is important to maintain robustness in the presence of confounding and selection bias (Prosperi et al., 2020; Sanchez et al., 2022). One way of representing known causal relationships among covariates is structural causal models, which can then be used to learn machine learning models that can support counterfactual evaluations such as Bayesian nets (Pawlowski et al., 2020). Causal directed acyclic graphs (DAGs) can also be learned from extensive observational data (Vowels et al., 2021). Combining the results of multiple causal structure learning algorithms with, e.g., majority voting, Mahipal and Alam (2022) would alleviate biases due to the different assumptions inherent in the algorithms. On the other hand, potential biases in the data, such as selection bias or not having common support for treatment and control cases in high-dimensional neighborhoods, can still negatively affect causal discovery and inference. In the case of Alzheimer's disease classification, Xia et al. (2022) augments the training data with counterfactuals of synthesized brain images from a pre-trained generative model using age as a conditional factor to alleviate spurious correlations and to improve classification performance. They use an adversarial game to identify the most useful counterfactual to add to the training data.

Identifying confounders and properly specifying the functional form are the challenges in causal discovery; hence, external evaluation of discovered relationships is necessary: For example, in the case of predicting pneumonia risk and 30-day hospital readmission, thanks to the interpretability of the GA^2^M model, a non-causal association was caught: the data implied that asthma was associated with a lower risk, which was actually due to the stricter treatment protocols for the patients with asthma (Caruana et al., 2015).

Several methods discover segments with different treatment effects. The qualitative interaction tree (QUINT) method (Dusseldorp and Van Mechelen, 2014) partitions patients into subgroups predicting which treatment works better for them based on the observed characteristics. The causal rule sets method finds short rules that do not necessarily have a tree structure (Wang and Rudin, 2022). The heterogeneous effect mixture model (HEMM) uses mixture distributions rather than crisp rules (Nagpal et al., 2020).

Causal machine learning models are differentiated from the predictive machine learning models in that they estimate the causal effect of treatment *w* on the outcome *y*, given other co-variates *X*, rather than predicting the outcome *y*, given *X* and *w*. Used with large amounts of data, and assuming that the data contain all confounders, these methods can discover heterogeneity dimensions and functional forms, and locally average over many potentially viable model structures and thus guard against confounding due to incorrect model specification.

A classification of the causal machine learning meta-learners aimed at learning the heterogeneous treatment effects considers three groups: T-learners learn the outcome for the treatment and control datasets separately and take the difference of the predicted values, S-learners use one learner that includes the treatment to predict the outcome and take the difference of the predicted outcome values with different treatment variable values keeping all else the same, X-learners (Künzel et al., 2019) offer the advantages for learning smooth or sparse HTE functions and when the sample sizes are unequal by fitting separate learners on the imputed treatment effects in the treatment and control groups and combining them with a weighted average.

Next, we provide a deeper perspective on the two causal machine learning methods we use in our study. Bayesian additive regression tree (BART) is a sophisticated S-learner, while causal forests (CF) do not fall within the meta-learner classification.

#### Causal machine learning

##### BART for treatment effects

Bayesian additive regression tree (BART) is a flexible sum-of-trees machine learning algorithm, which does not impose strong parametrization, provides uncertainty intervals, and allows for regularization through prior distributions (Chipman et al., 2010).

The BART model is the sum of many regression trees *f* (**x**) = *g*(**x**, *T*_1_,*M*_1_) + *g*(**x**,*T*_2_,*M*_2_)+· · ·+*g*(**x**,*T*_*m*_,*M*_*m*_), where *M*_*h*_ = (μ__*h*_1_,μ__*h*_2_,... μ_*hbh*_) denotes the collection of parameters for the *b*_*h*_ leaves of the *h*^th^ tree, *T*_*h*_. Unlike the parametric regression models, the BART model captures non-linearities and interactions without the explicit intervention of the researcher. To avoid overfitting, BART trees are designed to be small, while leaf parameters are shrunk toward zero. Each tree can be seen as a low-order interaction, which is particularly useful when the true function is also of low-order interaction. The trees are fit by posterior sampling and averaging, using the Bayesian backfitting and Markov chain Monte Carlo (MCMC) algorithms. The regularization prior distributions influence the size and fit (Hill et al., 2020); for example, when the data are high-dimensional and ultrasparse, using a sparsity-inducing Dirichlet prior filters out most of the variables unless the data strongly suggest otherwise (Linero, 2018). Chipman et al. (2006) found that BART maintained its strong performance with the default prior.

${\hat{\tau }}_{i}^{BART}$ is calculated as the mean conditional difference between the predicted outcome value under the treatment and control *w* levels, over the set of posterior samples ℂ.


$${\hat{\tau }}_{i}^{BART}=\sum_{\Omega \;\in \mathbb{C}}^{}\left(\left({\hat{y}}_{i}|X_{i},\;w_{i}=\omega_{s},\;\Omega \right)-\left({\hat{y}}_{i}|X_{i},\;w_{i}=\omega_{0},\;\Omega \right)\right)/\left|\mathbb{C}\right|$$

Hill (2011) remarks that BART provides “simultaneous inference on CATEs,^1^ sample average treatment effects, and everything in between.” Simulation experiments showed that BART had a higher accuracy in estimating nonlinear HTE than some other methods such as propensity score matching, propensity-weighted estimators and regression adjustments, and created coherent uncertainty intervals, which increased when there is less information (“lack of complete overlap and hence limited empirical counterfactuals”). Hill (2011) points out that BART's ability to deal with large numbers of predictors and discard unimportant ones is especially beneficial when the researcher is trying to avoid confounding bias and trying to satisfy ignorability by adding all possible covariates.

We use the BART R package implementation that supports continuous, as well as binary outcomes with probit and logistic BART (McCulloch, 2019), as one of the C-ML methods in the ensemble.

##### Causal forest

Causal forest (CF) builds on causal trees (Athey and Imbens, 2016) and the Random Forest algorithm (Breiman, 2001) to develop a non-parametric honest causal effect estimation capable of handling high dimensional data (Wager and Athey, 2018). The causal forest model has three design elements: honesty, local centering, and local weighting. Honesty refers to using separate subsets for model structure selection and estimation, which enables the use of asymptotic behavior assumption for the confidence intervals as if the model structure was exogenously given (Athey and Imbens, 2016). One subset is used for local centering of *w* and *y* to improve the finite-sample performance of estimates. Local centering regresses out the effect of features/covariates *x* from *w* and *y*, outputting residuals using leave-one-out estimates of the marginal expectation without the *i*-th observation. Athey et al. (2019) claims that by locally centering the outcomes before running the forest, the estimator is potentially robust to confounding factors, similar to the orthogonalization ideas discussed by Chernozhukov et al. (2018). Using the locally centered outcomes and treatment, they partition the *x* space into regions by building trees to get pure leaves in terms of the treatment effect, τ_*i*_. The second subset is used on these trees to find the local weights to estimate the treatment effects. Local weighting ensures that the heterogeneity of treatment effect is captured adaptively within the local space of each leaf.

In this paper, we use its *Generalized Random Forest* package implementation (Tibshirani et al., 2018; Athey et al., 2019) as one of the C-ML methods in the ensemble.

### Monotonicity and sign constraints

Sign constraint on linear least squares regression is referred to as non-negative least squares in the literature. Meinshausen (2013) and Slawski and Hein (2013) show that as a regularizer, sign constraints can provide the same convergence rate as the lasso, providing that the design matrix satisfies the positive eigenvalue condition, and the sign of coefficients is known based on prior knowledge. Koike and Tanoue (2019) extends the result to general convex loss functions and nonlinear response variables, including logistic regression.

Monotonicity constraints increase the interpretability of models (Hall and Gill, 2019). They can be used to relax the linearity assumption while providing the boundaries of the expected behavior. They reduce the search space in favor of a simpler shape that is in line with the domain knowledge.

It is well known that regularization in additive models, e.g., lasso and ridge regression, improves model performance in the presence of noise and the lack of large data, by biasing the coefficients in favor of fewer complex models. Monotonic constraints in non-parametric regression are termed isotonic regression; Guntuboyina and Sen (2018) studies the theoretical properties of isotonic regression and states that least squares and/or maximum likelihood can be used to obtain attractive theoretical and computational properties without additional explicit regularization but point to computational difficulties.

Traskin et al. (2013) use monotonicity constraints based on subject matter knowledge, for the effect of exposure (*w*) and its confounder interactions in logistic regression to estimate the population attributable fraction of disease due to a risk factor. They show in simulation studies that the impact is estimated substantially more accurately than without constraints.

Incorporating domain knowledge with monotonicity constraints has been shown to improve the accuracy of machine learning models in Bayesian networks (Altendorf et al., 2012) and with shape constraints in generalized additive models (Pya and Wood, 2015). Ali et al. (2020) empirically states that incorporating domain knowledge in Bayesian models with the sign and monotonicity constraints on the prior distribution (qualitative informative) rather than the traditional informative prior approach of setting the prior mean and standard deviation of the relevant model parameters leads to better models, i.e., with expected sign and monotonicity and holdout prediction performance.

### Explaining the black-box models

Research into methods to understand/explain black-box machine learning models has gained popularity due to the increasing use of machine learning models to make or support decisions (Goebel et al., 2018; Samek et al., 2019). Model interpretability is an important driver of model implementability since it (a) fosters impartiality in decision-making, by detecting and remedying bias in the training dataset, (b) highlights potential adversarial perturbations that could change the prediction, (c) and allows the domain experts to check that the model has an underlying truthful causality (Arrieta et al., 2020). Hence, establishing causality with models that are understandable by the domain experts, managers, and regulatory branch has been stated as an XAI goal.

Linear monotonic models are highly interpretable (Hall and Gill, 2019). Generalized linear models (GLMs) extend linear models to non-Gaussian responses within the exponential family, where *g* is the link function and can be the identity function, logit/probit, or log, for Gaussian, binomial or Poisson response, respectively.


$$\begin{array}{l} g\left(E\left(y|X\right)\right)=\alpha +\beta_{1}X_{1}+...+\beta_{p}X_{p} \end{array}$$

The generalized additive model (GAM) further extends the GLM framework to include smooth non-parametric functions *f*_*j*_ of the input variables, where the user specifies the degree of the spline function for each variable (Hastie and Tibshirani, 1990).


$$\begin{array}{l} g\left(E\left(y|X\right)\right)=\alpha +f_{1}\left(X_{1}\right)+..+f_{p}\left(X_{p}\right) \end{array}$$

Lou et al. (2012) introduces tree ensembles as *f*_*j*_(*X*_*j*_) (called shape functions) in addition to the regression splines. This increases the accuracy of the models but does not have the smoothness property. Generalized additive models plus interactions (GA^2^M) extend this work with the FAST algorithm to include two-way interaction terms ranked based on importance, which is particularly useful for high-dimensional datasets (Lou et al., 2013). We use the explainable boosting machine (EBM) in the InterpretML software (Nori et al., 2019), which provides a fast implementation of the GA^2^M algorithm to explain the black-box C-ML Ensemble.


$$\begin{array}{l} g\left(E\left(y|X\right)\right)=\alpha +\underset{j}{\sum }f_{j}\left(X_{j}\right)+\underset{k}{\sum }\underset{j}{\sum }f_{jk}\left(X_{j},X_{k}\right) \end{array}$$

## The problem and the methodology

We consider the situation where an intervention (e.g., an incentive for physical activity) can change *w*_*i*_, the level of treatment (e.g., physical activity^2^) for individual *i*, from ω_0_ to ω_*s*_ at a cost of *c*_*i*_, thereby affecting multiple outcomes *y*_*i, d*_ (e.g., diabetes and heart disease probability). The resulting benefit for outcome *d* and individual *i* is given by the heterogeneous treatment effect τ_*i, d*_. As defined earlier, τ_*i, d*_ = *E*[*y*_*i, d*_|_*w*_*i*_ = ω*s*_, *X*_*i*_]− *E*[*y*_*i, d*_|_*w*_*i*_ = ω0_, *X*_*i*_]; i.e., the change in the expected outcome when physical activity is increased from ω_0_ to ω_*s*_. We would like to maximize the total benefit by selecting which individuals to target with the intervention without exceeding the *budget*. Assuming that the benefits from multiple outcomes are additive, the optimization problem for targeting individuals subject to limited resources boils down to sorting the individual by their total benefit to cost ratio and going down the list until either the budget is used up or the marginal benefit to cost ratio falls below 1.

Next, we present a methodology for the challenging task of estimating τ_*i, d*_ justifiably, where we drop the subscript *d* for simplicity of exposition. The superscript will denote the estimation model.

### Overview of the methodology for HTE estimation

We would like to obtain stable (low variance) treatment effect estimates with an interpretable model that is consistent with the existing theory/domain knowledge and accurately captures the heterogeneity in treatment effects. The proposed methodology to achieve this goal has the following components.

- Causal machine learning ensemble (C-ML)
- Explainable AI (XAI)
- Generalized linear model with qualitative constraints (GLM_QC).

Causal machine learning ensemble discovers heterogeneity dimensions of the treatment effect, their functional shape, and interactions that may not be known or articulated in a theory-driven model specification. While the C-ML is expected to provide accurate estimates of the heterogeneous treatment effects with large-scale data that include all confounders, it is a black-box. XAI explains the C-ML in terms of an additive model, enabling the domain experts to evaluate whether the relationships make sense, and enabling the modeler to incorporate the relationships into the interpretable model specification. The novel GLM_QC method enables fast estimation of a generalized linear model, where nonlinear effects are modeled through discretized variables and sign and monotonicity constraints can be imposed on main and two-way interaction effects.

From the statistical learning perspective, C-ML works to reduce the bias, while regularization in the form of sign and monotonicity constraints in GLM_QC works to reduce the variance of the estimates. Having the domain expert and the modeler in the loop, deciding incrementally whether to modify the specification based on C-ML and XAI findings and the incremental improvement in targeting efficiency is an additional element that ensures complexity control.

**Figure 1:**
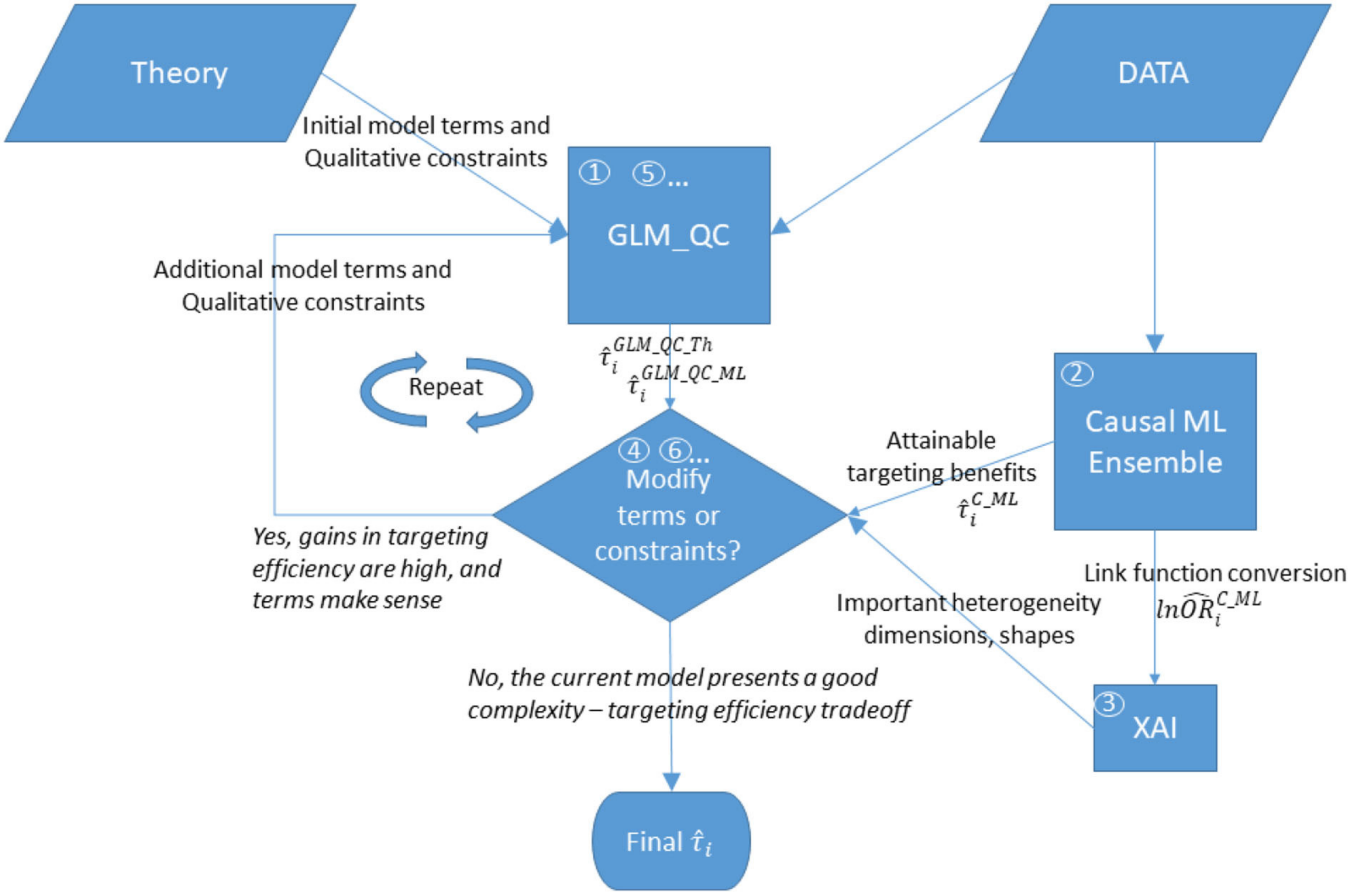
Overview of the proposed methodology. The numbers in circles represent the order of the steps, where boxes “GLM_QC” and “Modify terms or constraints?” are repeated until the modeler decides not to modify the model.

Figure 1 provides an overview of the proposed methodology. The steps are as follows:

(1) With large-scale observational data, GLM_QC fits the theory-driven generalized linear model specification, which models *g*(*E*(*y*)) as a function of *X* and *w*, allows nonlinearity, and includes qualitative constraints on the sign and monotonicity of the effects based on domain knowledge. This model is used to calculate the initial heterogeneous treatment effect estimates ${\hat{\tau }}_{i}^{GLM\text{\_}QC\text{\_}Th}\;$for the target population.
(2) The causal machine learning ensemble (C-ML Ensemble) independently provides completely data-driven HTE estimates, ${\hat{\tau }}_{i}^{C\text{\_}ML}$.
(3) To provide insights into important heterogeneity dimensions and their functional shape, the causal effect of *w* on *g*(*E*(*y*)) is explained with a global additive XAI tool, EBM.
(4) Based on the XAI explanation of the C-ML Ensemble, the most important heterogeneity dimension that is not represented in the current GLM_QC model or is represented with qualitative constraints that are inconsistent with the C-ML findings is identified as the next modification.
(5) The current GLM_QC model specification is updated with the identified modification, which can be an additional interaction effect with *w* and/or (modified) qualitative constraints. In the first iteration, GLM_QC_Th is updated and labeled GLM_QC_ML_1.
(6) The improvement in targeting efficiency due to the modification in the previous step is quantified. The modeler decides whether to keep the previous model or accept the modified model^3^ based on the credibility of the modified model considering extant knowledge/ theory, the added complexity, and the targeting efficiency improvement. Accordingly, either the next set of modifications is identified, or the previous model is declared final.

Steps 5 and 6 are repeated, incrementally updating the model until the modeler decides not to update the model anymore.

As mentioned earlier, we assume that (a) the treatment of an individual will not affect the outcome of others and the treatment *w* is standard and (b) that all confounding variables are observed and included in *X*. The outcome *y* can be binary or continuous, and the treatment *w* can be binary or ordinal.

Now, we provide the details about each component.

### GLM_QC

The proposed generalized linear model with qualitative constraint (GLM_QC) is a GLM (McCullagh and Nelder, 2019) subjected to sign and monotonicity restrictions on the main and two-way interaction effects specified by the modeler. GLM_QC models nonlinear effects by discretizing continuous variables, and unlike GAMs, GLM_QC does not require the function to be smooth but allows imposing increasing or decreasing monotonicity and sign constraints.

While monotonicity and sign constraints can be imposed on linear models using prior distributions, the estimation of these Bayesian models, especially with large datasets, is computationally intensive and may not converge (Ali et al., 2020). GLM_QC is fit with the efficient glmnet algorithm with well-established convergence properties (Friedman et al., 2010).

Whereas glmnet (Hastie and Qian, 2014) only allows sign constraints to be imposed on the coefficients, our reparameterization approach allows the estimation of a model with the sign and monotonicity constraints.

The GLM_QC model is as follows:


$$g\left(E\left(y|X\right)\right)=\alpha +\sum_{j}^{}X_{j}\beta_{j}+\sum_{(k,j)\in \;\chi }X_{j}\cdot X_{k}\gamma_{jk}$$

Here, *g* is the appropriate link function for the response variable *y* from an exponential family sampling distribution, which is the identity for a Gaussian response, and the logit for a Bernoulli response: $g\left(p\right)=ln\left(\frac{p}{1-p}\right)$ where *p* = *E*(*y* = 1|*X*). *X*_*j*_·*X*_*k*_ stands for elementwise multiplication of *X*_*j*_ and *X*_*k*_. β_*j*_ *and γ*_*jk*_ are coefficient vectors with matching dimensions. The specification includes the independent variables ${\tilde{X}}_{j}$, the set (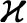) of two-way interactions, the sign constraints, the set Λ of variables where a non-parametric shape will be fit, and any constraints on their monotonicity. The continuous variables in the set Λ are discretized and coded with *K*_*j*_*-1* binary variables as follows, where ${\tilde{X}}_{j}\in \Lambda$ and takes values *1...K*_*j*._

$I_{j,k}=\{\begin{array}{c} 1,\;if\;{\tilde{X}}_{j}\;=k,\;for\;k\neq k_{j,ref}\\ 0\;otherwise \end{array}\;,where\;k_{j,ref}$ is the reference category.

The matrix X consists of *n*×1 column vectors $X_{j}={\tilde{X}}_{j}\;for\;{\tilde{X}}_{j}\notin \Lambda$, and *n*×*K*_*j*_−1 matrices $X_{j}=\left[\begin{array}{c} I_{j,1}...I_{j,K_{j}-1} \end{array}\right]$, for ${\tilde{X}}_{j}\in \Lambda$. This formulation, so far, allows the variables ${\tilde{X}}_{j}\in \Lambda$ to have arbitrary nonlinear shapes without monotonicity constraints.

#### Implementing the qualitative constraints with reparameterization

To impose the sign and monotonicity constraints, we reparametrize the model, as follows:


$$\begin{array}{l} g\left(E\left(y|Z\right)\right)=\alpha^{\prime }+\sum_{j}^{}Z_{j}\zeta_{j}+\sum_{(l,j)\in \;ℵ}Z_{l}\cdot Z_{j}\eta_{l,j}\\ \;Z_{j}=\{\begin{array}{c} X_{j}A_{j},\;for\;{\tilde{X}}_{j}\in \Lambda \\ X_{j}1,\;for\;{\tilde{X}}_{j}\notin \Lambda \end{array}\;, \end{array}$$

where *A*_*j*_ is a *K*_*j*_ – 1 × *K*_*j*_ – 1 invertible matrix, and *Z*_*j*_ is a variable of the reparametrized model. ζ_*j*_ and η_*l, j*_ are coefficient vectors for main and two-way effects, respectively. Table 1 provides the reparameterization for the main effect with coding variables. The “Monotonicity” and “Sign of the Effect” columns indicate the qualitative constraints that are to be imposed on the effect, and the next two columns indicate the coding variables in the reparametrized model and the sign restriction on their coefficients. The coding variables $\Delta_{jk}^{+}$ or $\Delta_{jk}^{-}$, represent the incremental effect of consecutive categories.


$$\begin{array}{l} \Delta_{jk}^{+}=\{\begin{array}{c} 1,\;if\;k\leq X_{j}\\ 0\;otherwise \end{array}\;,\;for\;k=1\ldots K_{j}-1\\ \;\Delta_{jk}^{-}=\{\begin{array}{c} 1,\;if\;k\geq X_{j}\\ 0\;otherwise \end{array}\;,\;for\;k=2...K_{j} \end{array}$$

$\Delta_{jk}^{+},\;and\;\Delta_{jk}^{-},\;$assume that the reference category *k*_*j, ref*_ is the last and first discretized value of the original variable, respectively. According to the coding variables, *A*_*j*_ is determined: it is a lower triangular matrix of 1 s for $\Delta_{jk}^{+},$ and an upper triangular matrix of 1 s for $\Delta_{jk}^{-}$.

**Table 1:** Coding monotonicity and sign constraints for discretized/ordinal variable *X*_*j*_.

**Case**	**Monotonicity of *X*_*j*_ effect**	**Sign of the effect**	**Coding variables**	**Constraints on coding variable coefficients**
1	Increasing	Positive	$\Delta_{jk}^{+},\;k=1...K_{j}-1$	≥0
2	Increasing	Negative	$\Delta_{jk,}^{-},\;k=2...K_{j}$	≤ 0
3	Decreasing	Positive	$\Delta_{jk,}^{-},\;k=2...K_{j}$	≥0
4	Decreasing	Negative	$\Delta_{jk}^{+},\;k=1...K_{j}-1$	≤ 0
5	Increasing	Unrestricted	$\Delta_{jk}^{+},\;k=1...K_{j}-1$	≥0, *for k*>1
6	Decreasing	Unrestricted	$\Delta_{jk}^{+},\;k=1...K_{j}-1$	≤ 0, *for k*>1

Note that a sign restriction without a monotonicity restriction can also be imposed on the effect of *X*_*j*_ categories relative to the reference category with sign constraints on the coefficients of the dummy variables *I*_*jk*_.

The reparametrized model with sign restrictions on the $\Delta_{jk}^{+},\;and\;\Delta_{jk}^{-},\;$is estimated with glmnet. The original model coefficients are recovered by multiplying with the coding matrix, e.g., β_*j*_ = *A*_*j*_ζ_*j*_.

Please see Appendices A, B in Supplementary material for reparameterization proofs of one and two-way interaction effects.

#### Estimating HTE with GLM_QC

To estimate HTE with GLM_QC, we use an S-learner approach (Künzel et al., 2019).


$$\begin{array}{l} {\hat{\tau }}_{i}^{GLM\_QC}=\;{\hat{g}}^{-1}\left(E\left(y|X,w=\omega_{s}\right)\right)-\\ \;{\hat{g}}^{-1}\left(E\left(y|X,w=\omega_{0}\right)\right) \end{array}$$

We assume that *X* contains all confounding covariates. The appropriate qualitative constraints are implemented as described above, treating *w* as any covariate.

If the outcome *y* is Gaussian, then *g*^−1^ is the identity and the heterogeneity of the treatment effect is determined only based on the interaction terms with *w*. But when the outcome is binary, *g*^−1^is the antilogit and nonlinear, all variables contribute to the heterogeneity of treatment effects.

### Causal machine learning ensemble

We construct the C-ML Ensemble by combining the predictions of two established but dissimilar causal ML methods: BART and causal forest (refer to Sections Heterogeneous treatment effect learning in healthcare and Causal machine learning); however, there is no restriction on the number or type of purely data-driven causal models that can be used in the ensemble. The C-ML Ensemble emphasizes consensus: the prediction is set to 0 unless the average of the independently fit ensemble component estimates is sufficiently away from 0, considering how well the component estimates agree for individual *i*, as follows.


$${\hat{\tau }}_{i}^{C\_ML}=\{\begin{array}{c} 0,\;if\;\left|\frac{{\hat{\tau }}_{i}^{BART}+{\hat{\tau }}_{i}^{CF}}{2}\right|\;\leq q\hat{\sigma_{i\;,}}\\ \frac{{\hat{\tau }}_{i}^{BART}+{\hat{\tau }}_{i}^{CF}}{2},\;otherwise \end{array}$$

where $\hat{\sigma_{i}}=|\frac{{\hat{\tau }}_{i}^{BART}-{\hat{\tau }}_{i}^{CF}}{2}|$ is the standard deviation of the average of the ensemble component estimates for this individual and *q* is a constant.

The causal forest models are implemented with the grf R package (Tibshirani et al., 2018; Athey et al., 2019), which provides predictions ${ŷ}_{i}^{CF},\;and\;{\hat{\tau }}_{i}^{CF}.\;$For ordinal causal variables, ${\hat{\tau }}_{i}^{CF}$ is constant across values of *w*, given *X*_*i*_.

The BART R package (McCulloch, 2019) is used for estimating ${\hat{\tau }}_{i}^{BART}$, which is the mean conditional difference between the predicted outcome value under the treatment and control *w* levels, over the set of posterior samples, refer to Section Heterogeneous treatment effect learning in healthcare. For binary outcomes, logit BART is used.

### Explaining the causal ML ensemble in terms of the link function

While the C-ML Ensemble directly predicts HTE, the GLM_QC models the outcome *y*, transformed by the link function, *g*(). To provide insights into important heterogeneity dimensions and their shape, the causal effect of *w* on *g*(*E*(*y*_*i*_)) is explained with a global additive XAI tool. Note that:


$$\begin{array}{l} g\left(E\left(y|X,w=\omega_{s}\right)\right)-g\left(E\left(y|X,w=\omega_{0}\right)\right)=\\ \;\{\begin{array}{c} {\hat{\tau }}_{i}^{C\_ML},\;if\;y\in \mathbb{R}\;\\ \ln{\hat{OR}}_{i}^{C\_ML},\;if\;y\;\in \left\{0,1\right\} \end{array} \end{array}$$

where $lnOR_{i}=ln\left(\frac{p_{i}|w_{i}=\omega_{s}}{1-p_{i}|w_{i}=\omega_{s}}/\frac{p_{i}|w_{i}=\omega_{0}}{1-p_{i}|w_{i}=\omega_{0}}\;\right)$.

When the link function is logit, to get insights into the causal machine learning ensemble model, we calculate the outcome log odds ratios for being treated vs. non-treated for each individual *i* using the C-ML Ensemble model predictions. This way they are directly comparable to the GLM_QC model coefficients for the treatment variable *w* and its interactions, as any logit model coefficient is interpreted as the log odds ratio associated with the respective effect.


$$\begin{array}{l} \ln{\hat{OR}}_{i}^{C\_ML}=\\ ln\left(\frac{{\hat{p}}_{i}^{C\_ML}|w_{i}=\omega_{s}}{1-{\hat{p}}_{i}^{C\_ML}|w_{i}=\omega_{s}}/\frac{{\hat{p}}_{i}^{C\_ML}|w_{i}=\omega_{0}}{1-{\hat{p}}_{i}^{C\_ML}|w_{i}=\omega_{0}}\right)\\ \;{\hat{p}}_{i}^{C\_ML}|w_{i}=0.5\;{\hat{p}}_{i}^{BART}|w_{i}+0.5\;{\hat{p}}_{i}^{CF}|w_{i},\\ \;{\hat{p}}_{i}^{BART}|w_{i}=\sum_{\Omega \;\in \mathbb{C}}^{}{\hat{P}}_{BART}\left(y_{i}=1|x_{i},w_{i},\Omega \right)/\left|\mathbb{C}\;\right|,\\ \;{\hat{p}}_{i}^{CF}|w_{i}={\hat{y}}_{i,OBS}^{CF}-(w_{i}-w_{i,OBS})\;{\hat{\tau }}_{i}^{CF} \end{array}$$

${ŷ}_{i,OBS}^{CF}$ and *w*_*i, OBS*_ stand for the actual observed *w* value for individual *i* and the CF predicted outcome at that value, respectively.

We use the EBM in the InterpretML software (Nori et al., 2019) to explain the ${\ln\hat{OR}}_{i}^{C\text{\_}ML}\;$globally in terms of an additive model of tree ensemble based shape functions with up to two-way interaction terms *f*_*j*_(*X*_*j*_) and *f*_*jk*_(*X*_*j*_, *X*_*k*_).


$$\begin{array}{l} g\left(E\left(y|X\right)\right)=\alpha +\underset{j}{\sum }f_{j}\left(X_{j}\right)+\underset{k}{\sum }\underset{j}{\sum }f_{jk}\left(X_{j},X_{k}\right) \end{array}$$

## The case

Non-communicable diseases such as diabetes and heart disease make up a significant portion of the burden of disease in the world (Ayer et al., 2014; Gakidou et al., 2017). The COVID-19 pandemic showed that they also induce severe complications when such communicable diseases strike (Guo et al., 2020; Khan et al., 2020). Employers and health insurance companies contract companies to engage their employees and customers in healthier lifestyles involving more exercise and healthy eating habits to reduce healthcare costs and improve quality of life (Fontil et al., 2016; Castro Sweet et al., 2018). Companies offer incentives to their employees to increase their physical activity, including economic incentives in motivational tools built into health wearables (Carrera et al., 2020; Adjerid et al., 2022).

Some interventions, such as providing accessible exercise facilities (Sallis et al., 1990), are directed to the population, while others can be targeted at the individual level, such as assigning personal coaches and providing incentives for attaining levels of activity as monitored by apps. Intervention cost resources and targeting resources on those who are most likely to benefit can maximize the benefit (reduction in disease burden) with a limited budget. On the other hand, decision-makers need to justify the allocation of resources to specific individuals or groups.

One of the objectives in the 2019–2023 Strategic Plan of the Turkish Ministry of Health is to increase the physical activity of the citizens, including individual incentives for achieving certain physical activity levels (strategy 1.2.2^4^). Governments around the world are implementing measures to increase physical activity (World Health Organization, 2019). Prior study showed that increasing physical activity has a substantial impact on decreasing diabetes and IHD prevalence (Ali et al., 2020). These two diseases constitute the fastest growing and the largest source of the burden of disease in the country, respectively.^5^ While global studies indicate that increasing physical activity decreases the probability of disease, the individual effects could vary due to demographic, socioeconomic, racial, and environmental differences.

In this case study, we estimate the HTE of increasing physical activity on diabetes and ischemic heart disease (IHD) probability among the adult population in Turkey. The case objective is to maximize the expected decrease in the prevalence of diabetes and IHD by targeting individuals with incentives for increasing their physical activity. We consider the benefit due to each disease to be equally important, and the cost of providing the incentive to be the same across individuals. We work with a fixed budget of resources sufficient for 25% of the population.

We use the proposed methodology to incorporate causal machine learning findings into theory-driven models, striking a reasonable balance between interpretability and efficiency. Data sources and the initial theory-driven model specification, including the sign and monotonicity constraints, are taken from Ali et al. (2020), which evaluates the impact of population-wide behavioral change scenarios including smoking, physical activity, and diet on the burden of disease in Turkey. Working within the Bayesian framework, this study shows that qualitative restrictions on the sign and monotonicity of the Bayesian model coefficients work better than informed prior distributions with parameters from theory.

The nationally representative biannual health surveys conducted by the Turkish Statistical Institute (TUIK) in the years 2008 to 2016 constitute the individual level data source with 93,528 observations for individuals 15 or older. The surveys provide self-reported socioeconomic, demographic, behavior, and disease information. More information about the surveys can be obtained from TUIK.^6^

The last wave from 2016 with 17,242 observations provides the most recent representative joint distribution of demographics, behaviors, and other covariates; hence, we use these observations with equal weighting when calculating the expected benefits. In this population, 18% of adults were inactive, 45% insufficiently active, 10% active, and 27% very active. The “very active” group is not targeted.

The model formulation in Ali et al. (2020) relies on large-scale meta-analysis results from Global Burden of Disease Studies (Gakidou et al., 2017). The logit of the probability that individual *i* has disease *d* is modeled separately for IHD and diabetes. Both models contain the following independent variables ${\tilde{X}}_{j}$: discretized (in Λ)*: Physical Activity, Age, Alcohol Consumption, Fruit Consumption, Vegetable Consumption, #Trusted individuals*. Binary: *Gender, Smoking, Second-hand Smoke exposure, Survey method*. Inherently categorical: *Region*. Linear: *Income Index, Education, BMI'*. Interaction affects in the IHD model are *Age* × *Gender, BMI'* × *Age, Smoke* × *Gender* × *Age, Alcohol* × *Gender, Physical Activity* × *Age*. Interaction affects in the Diabetes model are *Age* × *Gender, BMI'* × *Age, Smoke* × *Gender, Alcohol* × *Gender*.

Table 2 summarizes the qualitative constraints on the model coefficients: for example, the first line under the “Sign of the Effect” block indicates that all levels of physical activity (Insufficiently Active, Active, and Very Active) reduce disease risk relative to the Inactive, whereas the first line under the “Shape of the Effect” block indicates that the disease risk increases with age (not necessarily linearly)—all else being the same. These constraints are based on the 2016 GBD (Global Burden of Disease) meta-analysis results of hundreds of global studies on the relative risk (RR) of risk factors (BMI, Age, Gender), behaviors (Smoking, Secondhand Smoke, Physical Activity) for diabetes and IHD (Gakidou et al., 2017), as in Ali et al. (2020). Note that when the reference risk is low, RR is approximately equal to the odds ratio (Zhang and Kai, 1998), and thus, the appropriate ln(RR) sign and monotonicity can be used for the GLM_QC model coefficients.

**Table 2:** Qualitative constraints imposed on the qualitative informative hierarchical bayes models based on meta-analysis results of global studies.

	**IHD**	**Diabetes**
**Sign of the effect**		
Physical Activity (reference level inactive)	Negative	Negative
Smoking (reference level non-smoking)	Positive	Positive
Second-hand Smoke (reference level un-exposed)	Positive	Positive
BMI (reference level BMI < 22.5)	Positive	Positive
**Shape of effect**		
Male with Age	Increase	Increase
Female with Age	Increase	Increase
Physical Activity with Activity Level	Decrease	Decrease
Physical Activity with Age	Increase	NA
Male Smoking with Age	Decrease	NA
Female Smoking with Age	Decrease	NA
BMI with Age	Decrease	Decrease

Treatment, in this context, is one level increase in the individual's physical activity level (from 1 to 2, 2 to 3, and 3 to 4). Those who are already at the highest level of physical activity are not affected.


$$\tau_{i,d}=\{\begin{array}{c} E\left[y_{i,d}|\;PhysicalActivity_{i}=PA_{i}+1,X_{i}\right]-\\ \;E\left[y_{i,d}|PhysicalActivity_{i}=PA_{i},X_{i}\right],\;PA_{i}<4\\ 0,\;PA_{i}=4\; \end{array}$$

where *PA*_*i*_ is the observed physical activity of the individual.

## Results

All models are fit with the large-scale data and used to calculate ${\hat{\tau }}_{i}\;$(refer to Section The problem and the methodology) with the target population data represented by the last wave of surveys. A summary of the GLM_QC models that we developed following the proposed methodology is provided in the unshaded cells in Table 3. The shaded rows provide the Causal ML Ensemble models.

**Table 3:** Summary of the GLM_QC models and unconstrained benchmarks.

	**Model name**	**Physical activity interaction terms**	**Qualitative constraints**	**Benefit when targeting 25%**	**% lnOR positive**	**Benchmark GLM model**	**Benefit when targeting 25%**	**% lnOR positive**
Diabetes	GLM_QC_Th^Diab^	None	Theory-based: lnOR decreases monotonically with physical activity	65.0%	0	*GLM_Th^*Diab*^*	*69.2%*	*0%*
	GLM_QC_ML1^Diab^	+Smoking	Insight-based: Increasing physical activity has a more negative effect on smokers' lnOR than non-smokers	68.2%	0	*GLM_ML1^*Diab*^*	*68.6%*	*0%*
	GLM_QC_ML2^Diab^	+Age	Insight-based: Increasing physical activity has a more negative effect on lnOR as age increases	75.7%	0	*GLM_ML2^*Diab*^*	*72.0%*	*3%*
	GLM_QC_ML3^Diab^	+Trust	Insight-based: Increasing physical activity has a more negative effect on lnOR of those with more trusted friends	76.2%	0	*GLM_ML3^*Diab*^*	*73.0%*	*3%*
	GLM_QC_ML4^Diab^	+Activity × Second-hand Smoke	Insight-based: Increasing physical activity from inactive to insufficiently active has a smaller negative effect on lnOR in the presence of secondhand smoke; no monotonicity or sign constraint	76.6%	1%	*GLM_ML4^*Diab*^*	*75.0%*	*2%*
	*C_ML^*Diab*^*	*Black-box*		*78.5%*	*0*	
IHD	GLM_QC_Th^IHD^	Age	Theory-based: Increasing physical activity has a negative effect on lnOR but the effect becomes less negative as age increases	80.4%	0	*GLM_Th^*IHD*^*	*74.3%*	*23%*
	GLM_QC_ML1^IHD^	Age	Insight-based correction: Increasing physical activity has a negative effect on lnOR, and the effect becomes more negative as age increases	81.3%	0	*GLM_ML1^*IHD*^*	*74.3%*	*23%*
	GLM_QC_ML2^IHD^	+Education	Insight-based: Increasing physical activity has a negative effect on lnOR for those with more years of education, leveling at 8 years; implemented with min(education,8) interaction with activity coef > 0	80.9%	0	*GLM_ML_2^*IHD*^*	*76.3%*	*24%*
	*C_ML^*IHD*^*	*Black-box*		*84.0%*	*1%*	

**Figure 2:**
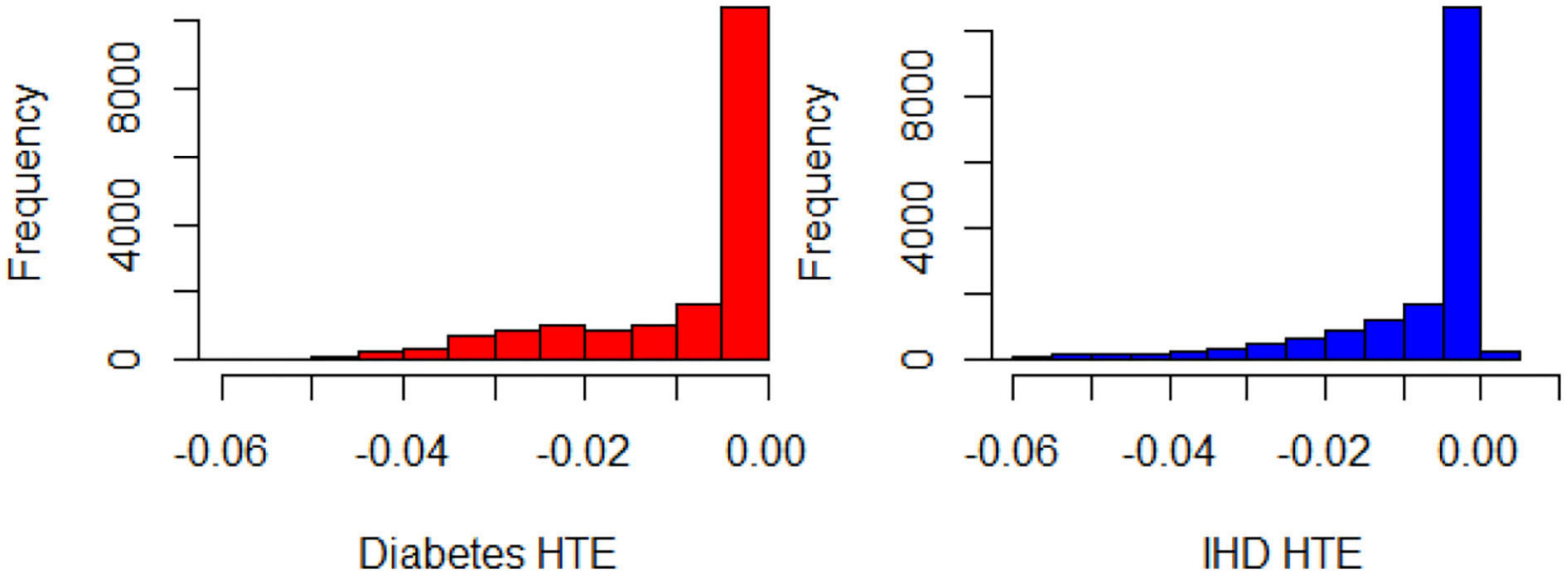
Histograms of ${\hat{\tau }}_{i}^{C\text{\_}ML}$ for Diabetes and IHD, all observations in the representative population (N = 17,242).

The initial GLM_QC model specifications are based on the literature (refer to the previous section). Causal forest and BART HTE estimates for the target population are combined as a causal machine learning ensemble, as explained in Section Causal machine learning ensemble, with *q* = √2. The two Causal ML algorithms agree very well on HTE predictions for diabetes, and reasonably well for IHD, with correlation coefficients of 0.92 and 0.79. The Causal ML Ensemble ${\hat{\tau }}_{i}^{C\text{\_}ML}$ distributions for the target population are provided in Figure 2.

Based on the extant theory, we would expect physical activity to reduce disease risk; hence, the distributions provide a face validity check. The “% ${\hat{\tau }}_{i,d}$ positive” column in Table 3 provides the percent of treatment effect predictions that are positive, which may be indicative of confounding problems. The initial theory-driven models for diabetes and IHD, GLM_QC_Th^Diab^ and GLM_QC_Th^IHD^ have all non-positive ${\hat{\tau }}_{i,d}$, as expected due to the sign constraints. It is remarkable; however, that the ${\hat{\tau }}_{i,Diab}^{C\text{\_}ML}$ is all non-positive, while 1% of ${\hat{\tau }}_{i,IHD}^{C\text{\_}ML}$ indicate a small increase in IHD probability with increased physical activity. By fitting complex interactions, nonlinear functions, and averaging over many model formulations, the Causal ML Ensemble avoids confounding bias due to incorrect model specification, as long as the confounding variables are observed in the dataset. Furthermore, using two dissimilar causal machine learning methods helps to avoid potential method bias.

The shaded columns in Table 3 provide a benchmark unconstrained GLM model for each GLM_QC model to assess the contribution of the qualitative constraints. We see that the GLM_Th^IHD^ model has the same terms as GLM_QC_Th^IHD^ but no constraints counterintuitively estimate that increasing physical activity increases the disease risk for 23% of the population. While the benchmark GLM_Th^Diab^ model has all negative HTE estimates, its coefficients imply that secondhand smoke decreases diabetes risk—which is counter to findings in the literature and may present a problem for the acceptance of the model. The benefit when targeting the 25% column in Table 3 provides the expected health benefit with a model *M* when the resource budget is set at 25% of the population. The benefit figures are scaled to read as a percent of the benefit that would be achievable if all individuals were targeted. The health benefit is calculated as the sum of the ${\hat{\tau }}_{i,d}^{C\text{\_}ML}$for the 25% individuals with the highest ${\hat{\tau }}_{i,d}^{M}$ . Here, we assume that the Causal ML Ensemble provides the true HTE, which is not unreasonable since our goal is to identify a model that emulates the C_ML model while remaining interpretable with respect to theory.

Note that with random targeting, we would expect to capture 25% of the national health benefits. Thus, all models provide much higher benefits than random targeting. For example, the theory-driven GLM_QC_Th^Diab^ and GLM_QC_Th^IHD^ capture 65.0 and 80.4% of the national attainable health benefit for diabetes and IHD, respectively. We compare these figures with the C_ML^Diab^ and C_ML^IHD^ performance to evaluate how much resource allocation (targeting) efficiency can be increased by incorporating insights from C_ML models: Diabetes targeting efficiency can be improved by up to 20% [calculated as (78.5–65.0%)/65.0% = 0.2)], while the potential gains for IHD are more modest at about 4%. Figure 7 provides the achieved health benefit in the target population with different models as well as random targeting and perfect targeting accomplished with the C_ML ensemble for comparison.

While these figures will change when targeting is based on the combined benefit from both diseases, they are useful for quantifying the performance–complexity tradeoff between models eliminating the noise due to multiple outcomes.

**Figure 3:**
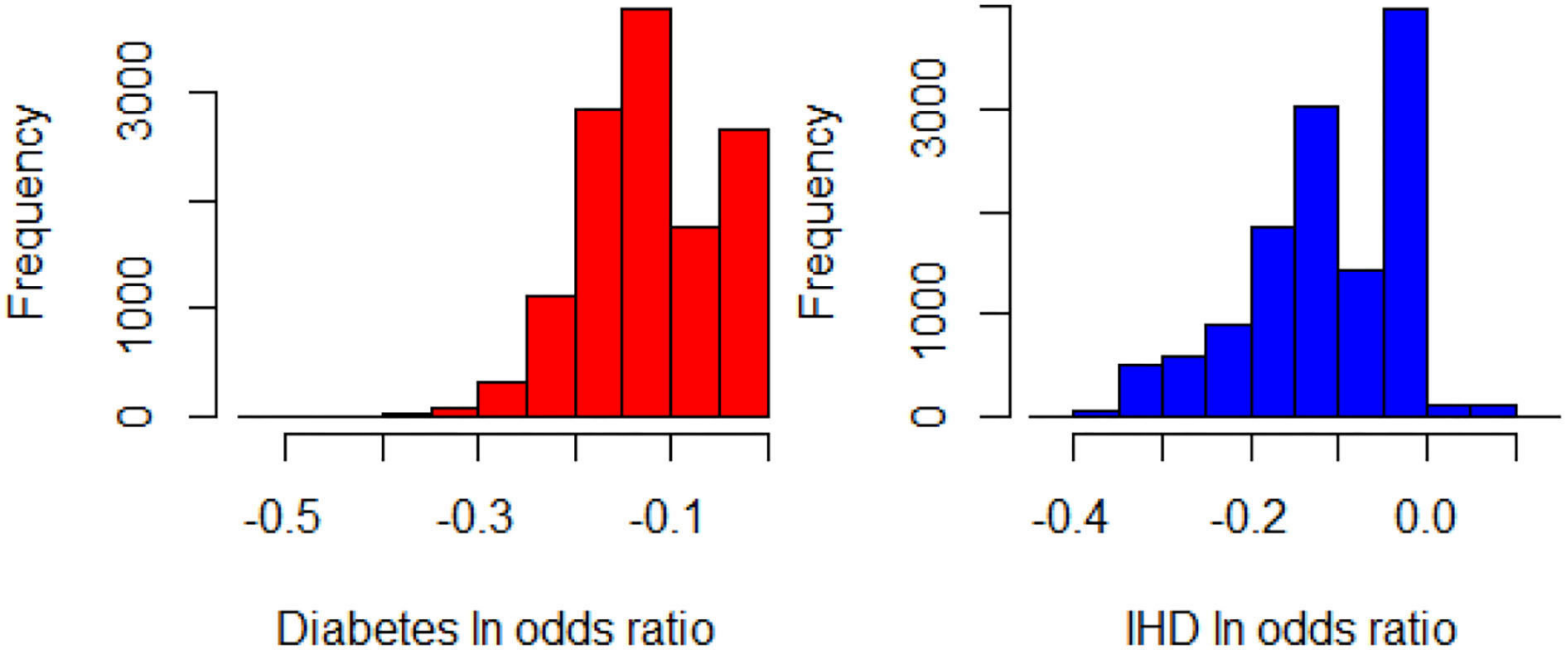
Histograms of ${\ln\hat{OR}}_{i}^{C\text{\_}ML}$for Diabetes and IHD, leaving out those individuals who are already Very Active in the target population (N = 12,580).

**Figure 4:**
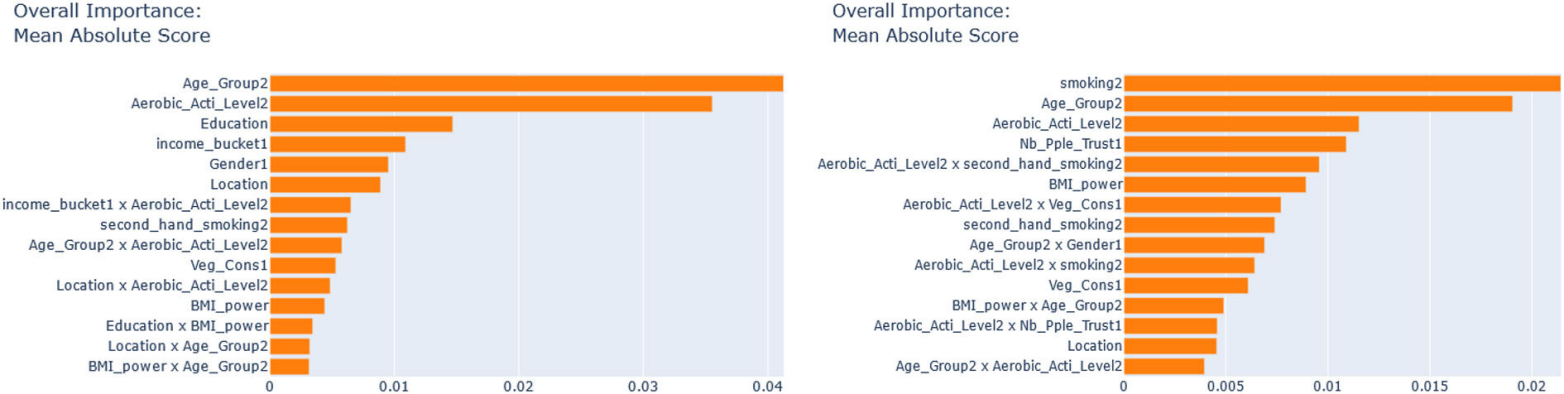
EBM feature importance explaining the ${\ln\hat{OR}}_{i}^{C\text{\_}ML}$. **(Left)** IHD, **(Right)** Diabetes.

**Figure 5:**
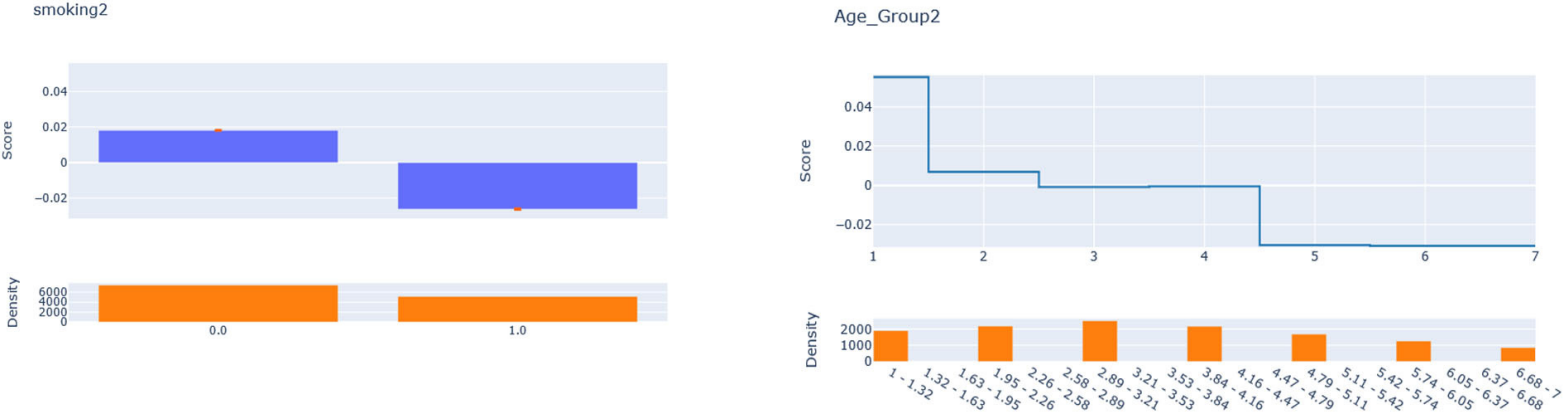
EBM additive terms for smoking and age explaining the diabetes ${\ln\hat{OR}}_{i}^{C\text{\_}ML}$.

**Figure 6:**
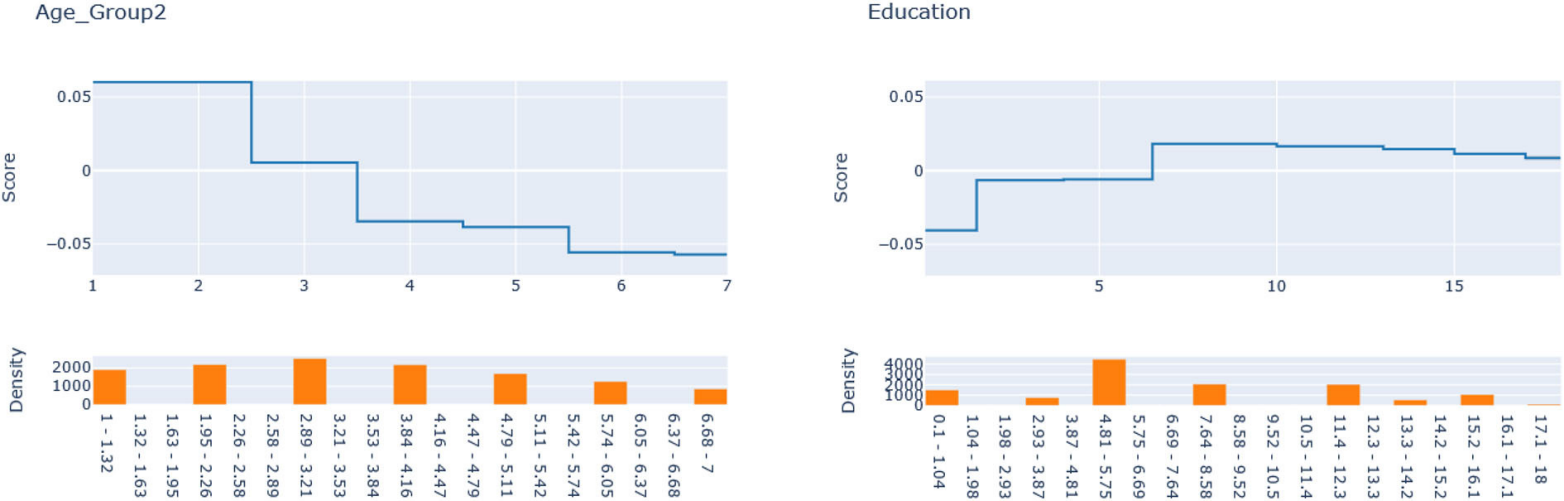
Explainable boosting machine (EBM) additive terms for Age and Education explain the IHD ${\ln\hat{OR}}_{i}^{C\text{\_}ML}$.

Next, we seek to get insights into the ${\ln\hat{OR}}_{i}^{C\text{\_}ML}$ to enhance the specification of the GLM_QC models. Figure 3 provides histograms of ${\ln\hat{OR}}_{i}^{C\text{\_}ML}$, leaving out those who are already Very Active in the target population and hence should not be targeted for increasing physical activity. We use the XAI model EBM to explain ${\ln\hat{OR}}_{i}^{C\text{\_}ML}$ for each disease. Figure 4 provides the overall importance of the additive terms according to the EBM. Figures 5, 6 visualize the most important terms in diabetes and IHD EBM models, respectively.

While the fidelity^7^ of these XAI models in terms of R^2^ is 0.86 for IHD and 0.62 for diabetes, these XAI models are only used to obtain insights, and the actual estimation is done through GLM_QC models.

We consider the major heterogeneity dimensions from Figure 4 for adding to the GLM_QC model as interaction effect with Physical Activity. We visualize the functional shape of the major effects for imposing monotonicity constraints.

According to Figure 4, for IHD, the top two contributors to the heterogeneity of ${\ln\hat{OR}}_{i}\;$are Age and Physical Activity level, followed distantly by Education. These two heterogeneity dimensions are already captured in the GLM_QC_Th^IHD^ model. However, as seen in Figure 6, the odds ratio decreases monotonically with age, whereas the theoretical specification was for it to increase. The GLM_QC_ML1^IHD^ model corrects the monotonicity constraint for the Age interaction, which improves the targeting efficiency, as seen in Table 3. The GLM_QC_ML2^IHD^ model adds the interaction term of Education with Physical Activity to incorporate the additional heterogeneity dimension for HTE. We choose to use GLM_QC_ML1^IHD^ for the final targeting since it is less complex, closer to theory, and has slightly better targeting efficiency.

As seen in Figure 4, Smoking, and Age group, followed by Physical Activity level, Number of People Trusted, and interaction of Physical Activity level with Second-hand Smoke are important heterogeneity dimensions of the odds ratio associated with increased Physical Activity and the individual's probability of having diabetes. As seen in Figure 5, the effect is more negative with Smoking and Decreases with age. Table 3 shows that the targeting efficiency increases with additional interaction terms and associated monotonicity and sign constraints being added to the GLM_QC_Th^IHD^ model; adding Smoking, Age, and Trust interactions (GLM_QC_ML3^IHD^) increases the expected health benefits when targeting 25% of the population to 76.2% of the nationally attainable benefits. While adding activity level-specific Second-hand Smoke terms to the model increase this figure to 76.6%, we choose to use the GLM_QC_ML3^IHD^ model, trading off marginal targeting efficiency gain for better interpretability. Note that with GLM_QC_ML3^IHD^, we achieved 97% of the benefits that we could achieve using the black-box C_ML^IHD^ model and obtained an interpretable model.

The discussed results focus on the heterogeneity of the ${\ln\hat{OR}}_{i}$ component of ${\hat{\tau }}_{i}$, which is represented in GLM_QC as interaction effects of *w*. All models have the same accuracy predicting, ${\hat{p}}_{i,0},$ the disease probability at the observed physical activity levels, with AUC in the target population: 0.77 for IHD and 0.82 for diabetes.

### The impact of qualitative constraints

**Figure 7:**
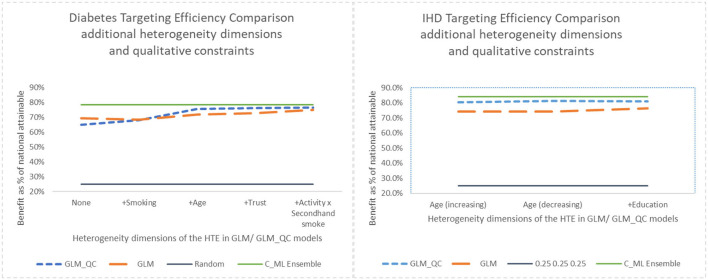
Benefit when targeting 25% of the population, adding heterogeneity dimensions with GLM_QC models compared to GLM models.

Next, we look at the contribution of the qualitative constraints. GLM_QC models result in better targeting efficiency than their unconstrained GLM benchmarks, as seen in Figure 7, with the exception of the GLM_Th^Diab^ model that—counter to theory based expectations—implies that secondhand smoke reduces the risk of diabetes. The GLM_QC_Th^Diab^ model coefficient for Second-hand Smoke is constrained to be non-negative based on the meta-analysis results that represent theory.

Adding interaction terms with the causal variable to the theoretical specification increases the targeting efficiency of both the GLM_QC and the benchmark GLM models. But the targeting efficiency of the GLM_QC models remains considerably higher than the GLM benchmarks even after adding terms.

Comparing the health benefits associated with the proposed methodology vs. using the traditional approach of taking the model terms from the literature and estimating with large-scale data (GLM_Th), we see an 11% improvement in diabetes and a 9% improvement in IHD benefits.

### Final targeting based on HTE estimates for both diseases

The total benefit due to targeting an individual is the sum of the ${\hat{\tau }}_{i,Diab}$ and ${\hat{\tau }}_{i,IHD}$, since we give each disease equal weight. The final GLM_QC_ML estimates capture 75.6 and 81.1% of the nationally attainable health benefits due to diabetes and IHD prevalence reduction, respectively. As expected, targeting to improve multiple outcomes results in somewhat lower benefits for each outcome compared to targeting solely to improve that specific outcome. Perfect targeting benchmarks using C_ML are 76.1% for Diab and 82.9% for IHD.

**Figure 8:**
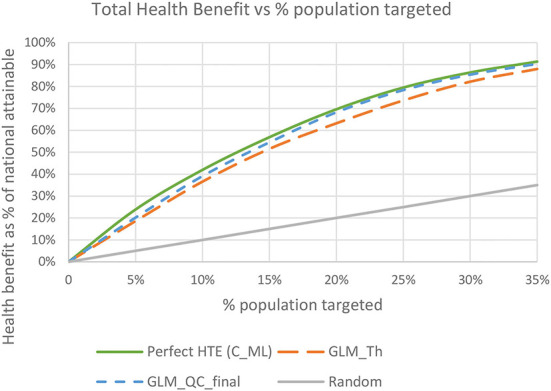
Total expected health benefit as a % of nationally attainable using the final GLM_QC_ML models.

Figure 8 shows the total expected national health benefits as a function of the percent of population targeted. We see that using models specified with terms from theory without qualitative constraints provides consistently lower benefits compared to following the proposed methodology regardless of the percent of the population targeted.

## Conclusions, managerial implications, and limitations

We have demonstrated a methodology to increase the benefits that can be obtained with the same resources by estimating the impact of the resource on each group/individual (HTE) accurately, without losing the connection to theory/domain knowledge for justifiable and efficient resource allocation decisions. Executives can generate more benefits with the same resources by leveraging big data and ML, without losing control over the process. Domain experts enhance their understanding of the effects of policies/interventions/treatments, by building on their existing knowledge, rather than using a black-box model—even if it is explained with XAI tools. This has implications for the acceptance of the resulting decisions.

The proposed methodology requires a summary of the current understanding of how the treatment affects the outcome, its important drivers, and the expected direction and monotonicity of the effects, along with large-scale data that contain these variables. Completely data-driven insights are identified with causal machine learning and explainable AI tools. These findings are successively incorporated into the theory-driven specification if the gain in expected benefits from more efficient resource allocation is deemed worth the additional model complexity and potential decrease in interpretability.

Central to the methodology is the GLM_QC method that we introduced to impose these sign and monotonicity constraints on main and two-way interaction effects with very short run times. The alternative Bayesian methods are computationally intensive and—especially with large datasets—may not converge.

The public health case study investigates targeting a quarter of the individuals in the Turkish adult population with individual incentives to increase physical activity to reduce the national disease burden caused by diabetes and heart disease. Compared to the traditional approach of using the model specification from the literature and estimating the model with large-scale data, the proposed methodology improves the expected health benefits for diabetes by 11% and for heart disease by 9%.

Experiments with the case-study data show that beyond improving interpretability, the regularization with qualitative constraints about the sign and monotonicity of the effect improves the HTE estimation and the resulting targeting efficiency.

The proposed methodology can be used in other domains where stakeholders need to be convinced of the rationale of the resource allocation decision, and large-scale data and domain knowledge are available. In particular, the healthcare industry can extend the current theory on the effect of treatments in different subpopulations, gaining additional insights into the drivers of effectiveness. These findings can also be used to design controlled trials in specific subpopulations.

A standard but important assumption of HTE estimation methods in general and the proposed methodology is that the observational data contain all confounding variables. Unobserved confounders, i.e., covariates that drive both the treatment decision and its effect on the outcome can bias the estimated treatment effects. The interpretability of the proposed models provides an important advantage in detecting potential confounding issues.

One of the limitations of the proposed methodology is that the XAI tool (EBM) relies on the additive models with low degree interaction effects for explaining the flexible black-box Causal ML Ensemble predictions and modeling the outcome. On the other hand, this should not be a big limitation based on prior research, which indicates that GAMs with up to two-way interaction effects can approximate many functions. In any case, the benchmark causal machine learning ensemble (C_ML) targeting performance informs the modeler about the additional benefits they are giving up for interpretability.

## Data availability statement

Publicly available datasets were analyzed in this study. This data can be found here: the dataset is available from TUIK by written request from the researcher to bilgi@tuik.gov.tr. The Turkish Statistical Institute does not allow sharing of this data by third parties.

## Author contributions

The author confirms being the sole contributor of this work and has approved it for publication.

## Footnotes

^1^CATE stands for conditional average treatment effect, another term for HTE.

^2^Other examples of treatment are a promotion in the marketing context, a communication with a specific message in the human resources context, or a change in the offered interest rate in the financial context.

^3^In the first iteration, the considered models are the GLM_QC_Th and the GLM_QC_ML_1, respectively.

^4^ https://stratejikplan.saglik.gov.tr/#

^5^ https://vizhub.healthdata.org/gbd-compare/

^6^ https://data.tuik.gov.tr/Bulten/Index?p=Turkey-Health-Survey-2016-24573

^7^Fidelity of an XAI model is the accuracy with which the XAI model (EBM) describes the AI model (C-ML) predictions.
