# Supplementary material for: Targeting resources efficiently and justifiably by combining causal machine learning and theory
Source: Front Artif Intell. 2022 Dec 7;5:1015604. doi: 10.3389/frai.2022.1015604 (PMC9768181; doi:10.3389/frai.2022.1015604)
Supplement: Supplementary file 1 [file Data_Sheet_1.docx]

**Targeting public health resources justifiably by combining causal machine learning and theory**

**Appendix A**

**One-way effect sign and monotonicity constraints with reparametrized variables**

Let

$I_{jk}=\left\{ \begin{matrix} 1, if X_{j}=k \\ 0 otherwise \end{matrix} \right.$ (1)

$\Delta_{jk}^{+}=\left\{ \begin{matrix} 1, if k\leq X_{j} \\ 0 otherwise \end{matrix} \right., for k=1..K-1$ (2)

$\Delta_{jk}^{-}=\left\{ \begin{matrix} 1, if k\geq X_{j} \\ 0 otherwise \end{matrix} \right., for k=2..K$ (3)

$\beta_{jk}$and $\gamma_{jk}$ are the coefficients of the model with the dummy variables and the re-parameterization.

Considering cases 1,4,5,6:

$\sum_{k} \beta_{jk}I_{jk}=\sum_{k=1}^{K-1} \gamma_{jk}\Delta_{jk}^{+}$ (4)

Let $X_{j}=k_{0}$, then $I_{jk_{0}}=1, I_{jk}=0 for k\neq k_{0}$, inserting in (4),

$\beta_{j,k_{0}}=\sum_{k=1}^{k_{0}} \gamma_{jk}, fork_{0}=1,..,K-1$ (5)

$\beta_{j,k_{0}}- \beta_{j{,k}_{0}-1}=\sum_{k=1}^{k_{0}} \gamma_{jk}-\sum_{k=1}^{k_{0}-1} \gamma_{jk}=\gamma_{j,k_{0}}, fork_{0}>1$ (6)

Case 1: since $\gamma_{jk}\geq0$, (5) and (6) are positive, hence the sign of the main effect is positive and increasing.

Case 4: since $\gamma_{jk}\leq0$, (5) and (6) are negative, hence the sign of the main effect is negative and decreasing.

Case 5: since $\gamma_{jk}\geq0, for k>1$, (6) is positive, hence the main effect is increasing, but (5) is unrestricted.

Case 6: since $\gamma_{jk}\leq0, for k>1$, (6) is negative, hence the main effect is decreasing, but (5) is unrestricted.

Considering cases 2 and 3:

$\sum_{k} \beta_{jk}I_{jk}=\sum_{k=2}^{K} \gamma_{jk}\Delta_{jk}^{-}$ (7)

Let $X_{j}=k_{0}$, then $I_{jk_{0}}=1, I_{jk}=0 for k\neq k_{0}$, inserting in (7),

$\beta_{j,k_{0}}=\sum_{k=k_{0}}^{K} \gamma_{jk}, fork_{0}=2,..,K$ (8)

$\beta_{j,k_{0}}- \beta_{j{,k}_{0}-1}=\sum_{k=k_{0}}^{K} \gamma_{jk}-\sum_{k=k_{0}-1}^{K} \gamma_{jk}={-\gamma}_{j,k_{0-1}}, fork_{0}>1$ (9)

Case 2: since $\gamma_{jk}\leq0$, (8) is negative, but (9) is positive, hence the sign of the main effect is negative and increasing.

Case 3: since $\gamma_{jk}\geq0$, (8) is positive, but (9) is negative, hence the sign of the main effect is positive and decreasing.

Sign and monotonicity constraints for a main effect are coded as follows.

| **Case** | **Monotonicity** | **Sign of the effect** | **Coding variables** | **Sign of coding variable coefficients** |
| --- | --- | --- | --- | --- |
| 1 | Increasing | Positive | $\Delta_{jk}^{+}, k=1..K-1$ | $\gamma_{jk}\geq0$ |
| 2 | Increasing | Negative | $\Delta_{jk,}^{-}, k=2..K$ | $\gamma_{jk}\leq0$ |
| 3 | Decreasing | Positive | $\Delta_{jk,}^{-}, k=2..K$ | $\gamma_{jk}\geq0$ |
| 4 | Decreasing | Negative | $\Delta_{jk}^{+}, k=1..K-1$ | $\gamma_{jk}\leq0$ |
| 5 | Increasing | Unrestricted | $\Delta_{jk}^{+}, k=1..K-1$ | $\gamma_{jk}\geq0, for k>1$ |
| 6 | Decreasing | Unrestricted | $\Delta_{jk}^{+}, k=1..K-1$ | $\gamma_{jk}\leq0, for k>1$ |

**Appendix B**

**Two-way effect sign and monotonicity constraints with reparametrized variables**

| **Case** | **Monotonicity of the** $X_{1}$ **effect** | **Monotonicity of** $X_{1}$ **interaction with** $X_{2}$ | **Sign of the total effect** | **Coding variables** | **Sign constraints on coding variable coefficients** |
| --- | --- | --- | --- | --- | --- |
| 7 | Increasing | Increasing | Positive | $\Delta_{{1k}_{1}}^{+}$, $\Delta_{{1k}_{1}}^{+}\boldsymbol{\times}\Delta_{{2k}_{2}}^{+}$,  $k_{1}=1..K_{1}-1,$  $k_{2}=1..K_{2}-1$ | $\gamma_{{1k}_{1}}\geq0, \gamma_{{1k}_{1},2k_{2}}^{'}\geq0$ |
| 8 | Increasing | Increasing | Negative | $\Delta_{{1k}_{1}}^{-}$, $\Delta_{{1k}_{1}}^{-}\boldsymbol{\times}\Delta_{{2k}_{2}}^{-}$,  $k_{1}=2..K_{1},$  $k_{2}=2..K_{2}$ | $\gamma_{{1k}_{1}}\leq0, \gamma_{{1k}_{1},2k_{2}}^{'}\leq0$ |
| 9 | Increasing | Decreasing | Positive | $\Delta_{{1k}_{1}}^{+}$, $\Delta_{{1k}_{1}}^{+}\boldsymbol{\times}\Delta_{{2k}_{2}}^{-}$,  $k_{1}=1..K_{1}-1,$  $k_{2}=2..K_{2}$ | $\gamma_{{1k}_{1}}\geq0, \gamma_{{1k}_{1},2k_{2}}^{'}\geq0$ |
| 10 | Increasing | Decreasing | Negative | $\Delta_{{1k}_{1}}^{-}$, $\Delta_{{1k}_{1}}^{-}\boldsymbol{\times}\Delta_{{2k}_{2}}^{+}$,  $k_{1}=2..K_{1},$  $k_{2}=1..K_{2}-1$ | $\gamma_{{1k}_{1}}\leq0, \gamma_{{1k}_{1},2k_{2}}^{'}\leq0$ |
| 11 | Decreasing | Increasing | Positive | $\Delta_{{1k}_{1}}^{-}$, $\Delta_{{1k}_{1}}^{-}\boldsymbol{\times}\Delta_{{2k}_{2}}^{+}$,  $k_{1}=2..K_{1},$  $k_{2}=1..K_{2}-1$ | $\gamma_{{1k}_{1}}\geq0, \gamma_{{1k}_{1},2k_{2}}^{'}\geq0$ |
| 12 | Decreasing | Increasing | Negative | $\Delta_{{1k}_{1}}^{+}$, $\Delta_{{1k}_{1}}^{+}\boldsymbol{\times}\Delta_{{2k}_{2}}^{-}$,  $k_{1}=1..K_{1}-1,$  $k_{2}=2..K_{2}$ | $\gamma_{{1k}_{1}}\leq0, \gamma_{{1k}_{1},2k_{2}}^{'}\leq0$ |
| 13 | Decreasing | Decreasing | Positive | $\Delta_{{1k}_{1}}^{-}$, $\Delta_{{1k}_{1}}^{-}\boldsymbol{\times}\Delta_{{2k}_{2}}^{-}$,  $k_{1}=2..K_{1},$  $k_{2}=2..K_{2}$ | $\gamma_{{1k}_{1}}\geq0, \gamma_{{1k}_{1},2k_{2}}^{'}\geq0$ |
| 14 | Decreasing | Decreasing | Negative | $\Delta_{{1k}_{1}}^{+}$, $\Delta_{{1k}_{1}}^{+}\boldsymbol{\times}\Delta_{{2k}_{2}}^{+}$,  $k_{1}=1..K_{1}-1,$  $k_{2}=1..K_{2}-1$ | $\gamma_{{1k}_{1}}\leq0, \gamma_{{1k}_{1},2k_{2}}^{'}\leq0$ |
| 15 | Increasing | Increasing | Unrestricted | $\Delta_{{1k}_{1}}^{+}$, $\Delta_{{1k}_{1}}^{+}\boldsymbol{\times}\Delta_{{2k}_{2}}^{+}$ | $\geq0, \forall$ $k_{1}\geq2, \forall k_{2}$ |
| 16 | Increasing | Decreasing | Unrestricted | $\Delta_{{1k}_{1}}^{+}$, $\Delta_{{1k}_{1}}^{+}\boldsymbol{\times}\Delta_{{2k}_{2}}^{-}$ | $\geq0, \forall k_{1}\geq2, \forall k_{2}$ |
| 17 | Decreasing | Increasing | Unrestricted | $\Delta_{{1k}_{1}}^{+}$, $\Delta_{{1k}_{1}}^{+}\boldsymbol{\times}\Delta_{{2k}_{2}}^{-}$ | $\geq0, \forall k_{1}\geq2, \forall k_{2}$ |
| 18 | Decreasing | Decreasing | Unrestricted | $\Delta_{{1k}_{1}}^{+}$, $\Delta_{{1k}_{1}}^{+}\boldsymbol{\times}\Delta_{{2k}_{2}}^{+}$ | $\leq0, \forall$ $k_{1}\geq2, \forall k_{2}$ |

$\beta_{{1k}_{1}}, \beta_{{1k}_{1},{1k}_{1}}^{'}$and $\gamma_{{1k}_{1}}, \gamma_{{1k}_{1},2k_{2}}^{'}$are the main effect and interaction effect coefficients of the model with the dummy variables and the re-parameterization.

Considering cases 7 and 14:

$\sum_{k_{1}} \beta_{{1k}_{1}}I_{{1k}_{1}}+\sum_{k_{1}} \sum_{k_{2}} \beta_{{1k}_{1},2k_{2}}^{'}I_{{1k}_{1}}I_{2k_{2}}=\sum_{k_{1}=1}^{K_{1}-1} \gamma_{{1k}_{1}}\Delta_{{1k}_{1}}^{+}+\sum_{k_{1}=1}^{K_{1}-1} \sum_{k_{1}=1}^{K_{1}-1} \gamma_{{1k}_{1},2k_{2}}^{'}\Delta_{{1k}_{1}}^{+}\Delta_{2k_{2}}^{+}$ (10)

**Sign of the total effect**

Let ${X_{1}=w_{0}, and X}_{2}=k_{0}$, then $I_{{1k}_{1}}=1 for k_{1}=w_{0}$, and 0 otherwise, and $I_{2k_{2}}=1, for k_{2}=k_{0}$ , 0 otherwise; inserting in (10) and simplifying,

$\beta_{{1w}_{0}}+\beta_{{1w}_{0},2k_{0}}^{'}$ =$\sum_{k_{1}=1}^{w_{0}} \gamma_{{1k}_{1}}+ \sum_{k_{1}=1}^{w_{0}} \sum_{2=1}^{k_{0}} \gamma_{{1k}_{1},2k_{2}}^{'},$ $forw_{0}=1,..,K_{1}-1, k_{0}=1,..,K_{2}-1$(11)

**Monotonicity of the** $X_{1}$ **effect**

$\beta_{{1w}_{0}}+\beta_{{1w}_{0},2k_{0}}^{'} - \left( \beta_{{1,w}_{0-1}}+\beta_{{1w}_{0}-1,2k_{0}}^{'} \right)$=$\sum_{k_{1}=1}^{w_{0}} \gamma_{{1k}_{1}}+ \sum_{k_{1}=1}^{w_{0}} \sum_{k_{2}=1}^{k_{0}} \gamma_{{1k}_{1},2k_{2}}^{'}-(\sum_{k_{1}=1}^{w_{0-1}} \gamma_{{1k}_{1}}+ \sum_{k_{1}=1}^{w_{0-1}} \sum_{k_{2}=1}^{k_{0}} \gamma_{{1k}_{1},2k_{2}}^{'})$= $\gamma_{{1w}_{0}}+\gamma_{{1w}_{0},2k_{0}}^{'}$ (12)

**Monotonicity of** $X_{1}$ **interaction with** $X_{2}$

$\beta_{{1w}_{0}}+\beta_{{1w}_{0},2k_{0}}^{'} - \left( \beta_{{1,w}_{0}}+\beta_{{1w}_{0},2k_{0}-1}^{'} \right)$=$\sum_{k_{1}=1}^{w_{0}} \gamma_{{1k}_{1}}+ \sum_{k_{1}=1}^{w_{0}} \sum_{k_{2}=1}^{k_{0}} \gamma_{{1k}_{1},2k_{2}}^{'}-\left( \sum_{k_{1}=1}^{w_{0}} \gamma_{{1k}_{1}}+ \sum_{k_{1}=1}^{w_{0}} \sum_{k_{2}=1}^{k_{0}-1} \gamma_{{1k}_{1},2k_{2}}^{'} \right)$= $\gamma_{{1w}_{0},2k_{0}}^{'}$ (13)

Case 7: since $\gamma_{{1k}_{1}},\gamma_{{1k}_{1},2k_{2}}^{'}\geq0$, it follows that (11), (12), (13) are positive.

Case 14: since $\gamma_{{1k}_{1}},\gamma_{{1k}_{1},2k_{2}}^{'}\leq0$, it follows that (11), (12), (13) are negative.

Cases 15 through 18 are similar to Cases 5 and 6.
